# Supplementary material for: The genome sequence of a hoverfly, Cheilosia impressa (Loew, 1840)
Source: Wellcome Open Res. 2024 Feb 19;9:74. [Version 1] doi: 10.12688/wellcomeopenres.20624.1 (PMC11255551; doi:10.12688/wellcomeopenres.20624.1)
Supplement: Peer review history

# Reviewer report: Reviewer response for version 1

**DOI:** 10.21956/wellcomeopenres.22827.r87592

**Published:** 2024-07-17

**Authors:** Jerome H L Hui

**Affiliations:** 1 The Chinese University of Hong Kong, Hong Kong, Hong Kong

In this Data Note, Falk and colleagues sequenced and assembled the genome of hoverfly *Cheilosia impressa* (Loew, 1840). Occurrence of this species has been recorded in England, Wales and Scotland. Molecular data of this species are scarce prior to this report, and are mainly mitochondrial cytochrome c oxidase subunit I (COI) gene sequences deposited to the NCBI database. Therefore, this new genome resource will be useful for further studies, ranging from insect-plant interaction, its population structure, and their evolution with other insects.

 This genome resource is excellent from the summary statistics, with high BUSCO number scores, high sequence continuity, and majority of sequences contained on the 5 pseudochromosomes (plus sex chromosome and mitochondrion). All in all, this is another valuable contribution.

Are sufficient details of methods and materials provided to allow replication by others?

Yes

Is the rationale for creating the dataset(s) clearly described?

Yes

Are the datasets clearly presented in a useable and accessible format?

Yes

Are the protocols appropriate and is the work technically sound?

Yes

Reviewer Expertise:

Genomics, evolution, invertebrates

I confirm that I have read this submission and believe that I have an appropriate level of expertise to confirm that it is of an acceptable scientific standard.

# Reviewer report: Reviewer response for version 1

**DOI:** 10.21956/wellcomeopenres.22827.r76539

**Published:** 2024-04-12

**Authors:** Annabel Whibley

**Affiliations:** 1 The University of Auckland, Auckland, Auckland, New Zealand; 2 Bragato Research Institute, Blenheim, New Zealand

Falk, Poole and colleagues describe a high-quality chromosome-level assembly of the hoverfly *Cheilosia impressa*  produced following protocols, workflows and reporting templates developed by the DToL consortium.  The resulting assembly has excellent quality parameters and will be a useful community resource.

**Minor comments**

 This is not the first DToL assembly report that I have seen with a k-mer completeness of 100% and I find this somewhat counter-intuitive when the report describes a primary assembly and also states that alt contigs are reported and desposited. The k-mer completeness is calculated as the fraction of reliable k-mers in the read set that are also found in the assembly. But I would expect that the primary assembly would show some degree of incompleteness due to kmers unique to the alt contigs. Or does the completeness consider both primary and alt contigs together (in which case this should be specified)? 

 Abstract: “Most of the assembly is scaffolded…” I think it would be more informative to also state the percentage of the assembly that has been assigned to chromosomes in this short abstract.

Are sufficient details of methods and materials provided to allow replication by others?

Yes

Is the rationale for creating the dataset(s) clearly described?

Yes

Are the datasets clearly presented in a useable and accessible format?

Yes

Are the protocols appropriate and is the work technically sound?

Yes

Reviewer Expertise:

Genomics Bioinformatics Evolution

I confirm that I have read this submission and believe that I have an appropriate level of expertise to confirm that it is of an acceptable scientific standard.
